# Prevalence of contraindications to mefloquine use among USA military personnel deployed to Afghanistan

**DOI:** 10.1186/1475-2875-7-30

**Published:** 2008-02-11

**Authors:** Remington L Nevin, Paul P Pietrusiak, Jennifer B Caci

**Affiliations:** 1 Army Medical Surveillance Activity, 2900 Linden Lane, Suite 200, Silver Spring, MD 20910, USA; 2 US Army Center for Health Promotion and Preventive Medicine, APG, MD 21010, USA; 3 Headquarters, 82^nd ^Airborne Division, Ft. Bragg, NC 28310, USA

## Abstract

**Background:**

Mefloquine has historically been considered safe and well-tolerated for long-term malaria chemoprophylaxis, but its prescribing requires careful attention to rule out contraindications to its use, including a history of certain psychiatric and neurological disorders. The prevalence of these disorders has not been defined in cohorts of U.S. military personnel deployed to areas where long-term malaria chemoprophylaxis is indicated.

**Methods:**

Military medical surveillance and pharmacosurveillance databases were utilized to identify contraindications to mefloquine use among a cohort of 11,725 active duty U.S. military personnel recently deployed to Afghanistan.

**Results:**

A total of 9.6% of the cohort had evidence of a contraindication. Females were more than twice as likely as males to have a contraindication (OR = 2.48, P < 0.001).

**Conclusion:**

These findings underscore the importance of proper systematic screening prior to prescribing and dispensing mefloquine, and the need to provide alternatives to mefloquine suitable for long-term administration among deployed U.S. military personnel.

## Background

Malaria poses a continued threat to U.S. military personnel. At least 423 blood-smear confirmed cases of malaria were diagnosed among members of the U.S. military between January 1st, 2000 and December 31st, 2005; of which at least 64 represent cases attributable to service in Afghanistan [1]. Outbreaks of malaria among U.S. military personnel attributable to service in Afghanistan are well described [1,2], of which infection due to *Plasmodium vivax *is the principal cause. To protect against the threat of malaria, U.S. military personnel deploying to Afghanistan may be prescribed mefloquine, which must be taken continuously throughout deployments lasting as long as 15 months. Although the long-term use of mefloquine for malaria chemoprophylaxis has historically been considered safe and well-tolerated among civilian travelers [3,4] and deployed military personnel [5], careful prescribing is needed to minimize the potential for severe neuropsychiatric adverse events, which may include acute psychoses, anxiety, depression, paranoia, myoclonus, and seizures [5]. Although the underlying mechanism of these adverse events is unknown, individuals with certain psychiatric and neurological histories appear to be at highest risk [4,5]. The U.S. package insert cautions that mefloquine "should not be prescribed for prophylaxis in patients with active depression, a recent history of depression, generalized anxiety disorder, psychosis, or schizophrenia or other major psychiatric disorders, or with a history of convulsions" [6].

To quantify the prevalence of, and identify demographic characteristics associated with contraindications to mefloquine use among deployed U.S. military personnel, a retrospective cohort study was performed using information available in military personnel, medical surveillance [1,4,7], and pharmacosurveillance databases [4,8].

## Methods

### Study population

To identify the study cohort, a roster was obtained comprising all active duty U.S. military personnel assigned in support of combat and reconstruction operations in Afghanistan and deployed within six months of a reference date in early 2007. Demographic covariate data on each was obtained from the Defense Medical Surveillance System (DMSS) [7], to include age category (as of the reference date), race (categorized as black, white, and other), gender, number of prior deployments, and military specialty (categorized as combat, and non-combat according to methods previously described) [5].

### Medical contraindications

For each member of the study cohort, DMSS was queried for International Classification of Diseases, Ninth Revision, Clinical Modification (ICD-9CM) -coded [9] diagnoses from inpatient and outpatient visits occurring within the 365 days prior to the date of deployment, consistent with psychiatric and neurological contraindications to mefloquine use [6]. These included depression, generalized anxiety disorder, psychosis, schizophrenia, and other major psychiatric disorders, which were defined to include bipolar disorders, obsessive-compulsive disorders, panic disorders, attention-deficit and attention-deficit hyperactivity disorders (ADHD), dissociative, conversion and factitious disorders, delusional disorders, and post-traumatic stress disorder. Also included as indicative of depression were adjustment disorders with depressed mood, dysthymic disorders, and cyclothymic personality disorder. Specifically excluded were drug and alcohol-related disorders; transient, acute or poorly-defined conditions; non-specific phobic disorders and non-depressive personality disorders, sexual and gender identity disorders, and somatoform diagnoses. Specific neurological diagnoses consistent with a liberal interpretation of a history of convulsions were included, to include Parkinson's disease, extrapyramidal diseases and other movement disorders, and epilepsy. A full list of diagnoses used to define a medical contraindication is provided as Table 1.

**Table 1:** Medical diagnoses consistent with contraindication to mefloquine use.

**Diagnosis**	**ICD-9CM Codes**
Major depressive disorder	296.2–296.3
Adjustment disorder with depressed mood	309.0, 309.28
Prolonged depressive reaction	309.1
Dysthymic disorder	300.4
Depression	311
Cyclothymic disorder	301.13
Generalized anxiety disorder	300.02
Psychoses (non-organic)	298
Schizophrenia	295
Bipolar and manic disorders	296.0, 296.1, 296.3–296.8
Obsessive-compulsive disorder	300.3
Panic disorder (with and without agorophobia)	300.01, 300.21
Attention-deficit disorders (with or without hyperactivity)	314.0
Dissociative, conversion and factitious disorders	300.1
Delusional disorders	297
Post-traumatic stress disorder	309.81
Parkinsonism	332
Extrapyramidal diseases and movement disorders	333
Epilepsy	345

### Pharmacologic contraindications

For each member of the study cohort, Pharmacy Data Transaction System (PDTS) records [8] were examined for prescription drugs dispensed at military, retail and mail-order pharmacies within the 180 days prior to deployment, whose use would be consistent with the treatment of psychiatric and neurological contraindications. These prescription drugs included ADHD treatments, antipsychotics, anticonvulsants, antiparkinsonians, sedative-anxiolytics, and antidepressants. Specifically excluded were amantadine, antidepressants commonly prescribed for smoking cessation (buproprion), as were the short-acting hypnotics commonly prescribed as sleep-aids. A full list of prescription drugs used to define a pharmacologic contraindication is provided as Table 2.

**Table 2:** Prescription drugs consistent with a contraindication to mefloquine use

**ADHD Treatments**
Amphetamines, atomoxetine, methyphenidate, modafinil
**Anticonvulsants**
Carbamazepine, clonazepam, divalproex sodium, gabapentin, lamotrigine, levetiracetam, oxcarbazepim, phenytoin, pregabalin, topiramate
**Antidepressants**
Citalopram, duloxetine, escitalopram, fluoxetine, flurazepam, mirtazapine, nefazodone, nortriptyline, paroxetine, sertraline, venlafexine
**Antiparkinsonians**
Bromocriptine, ropinirole
**Antipsychotics**
Aripiprazole, haloperidol, olanzapine, olanzapine/fluoxetine, quetiapine, risperidone
**Sedatives – anxiolytics**
Alprazolam, buspirone, chlordiazepoxide, diazepam, lorazepam, oxazepam, temazepam, trazodone, triazolam

### Statistical methods

Analyses included producing summary and descriptive statistics. The prevalence and the statistical significance of associations between demographic characteristics and a contraindication to mefloquine use were determined. All statistical analyses, including producing Pearson's χ^2^ statistics, P-values, calculation of crude odds ratios (ORs) and associated 95% confidence intervals (CIs) were performed using SAS software (Version 9.1; SAS Institute, Cary, NC) [10].

## Results

The study cohort consisted of 11,725 subjects. A total of 558 subjects (4.8%) had a medical contraindication (i.e. one or more psychiatric or neurological diagnoses prior to deployment consistent with a contraindication to mefloquine use). A total of 837 (7.1%) had a pharmacologic contraindication (i.e. one or more prescriptions filled prior to deployment for medications consistent with a contraindication to mefloquine use). A total of 1,127 (9.6%) had either or both (i.e. at least one or more such diagnosis or at least one or more prescription) (Table 3), comprising 916 males (8.6%) and 211 females (19.0%).

**Table 3:** Demographics of the study cohort and those with a medical or pharmacologic contraindication to mefloquine use, U.S. military personnel deployed to Afghanistan, 2007.

		**Contraindication(s) to mefloquine**		
	**Study cohort**	**Medical**	**Pharmacologic**	**Either or Both**
	Number (%)	Number (%)	Number (%)	Number (%)
**Total**	11725 (100)	558 (100)	837 (100)	1127 (100)
**Gender**				
Male	10614 (90.5)	463 (83.0)	670 (80.0)	916 (81.3)
Female	1111 (9.5)	95 (17.0)	167 (20.0)	211 (18.7)
**Race**				
White	8552 (72.9)	428 (76.7)	626 (74.8)	846 (75.1)
Black	1967 (16.8)	84 (15.1)	118 (14.1)	171 (15.2)
Other	1206 (10.3)	46 (8.2)	93 (11.1)	110 (9.8)
**Prior deployments**				
0	7119 (60.7)	315 (56.5)	494 (59.0)	660 (58.6)
1	3442 (29.4)	175 (31.4)	256 (30.6)	347 (30.8)
2+	1164 (9.9)	68 (12.2)	87 (10.4)	120 (10.6)
**Military specialty**				
Combat	2818 (24.0)	112 (20.1)	153 (18.3)	214 (19.0)
Non-combat	8907 (76.0)	446 (79.9)	684 (81.7)	913 (81.0)
**Age**				
18–19	521 (4.4)	27 (4.8)	18 (2.2)	38 (3.4)
20–29	7701 (65.7)	356 (63.8)	508 (60.7)	702 (62.3)
30–39	2774 (23.7)	138 (24.7)	240 (28.7)	301 (26.7)
40–49	688 (5.9)	37 (6.6)	66 (7.9)	81 (7.2)
50+	41 (0.3)	0 (0.0)	5 (0.6)	5 (0.4)

Of the 558 total with one or more diagnoses, 27 (4.8%) had neurological diagnoses and 531 (95.2%) had psychiatric diagnoses. The groups were mutually exclusive. Among those with neurological diagnoses (0.2% of the study cohort), all 27 had outpatient diagnoses, and none had inpatient diagnoses. Of the 27, 13 (48%) had primary diagnoses. Among those with psychiatric diagnoses (4.5% of the study cohort), three had inpatient diagnoses, and the remainder had one or more outpatient diagnoses, of which 411 (78%) had primary diagnoses.

Of the 837 with one or more prescriptions, 78 (9.4%) had a prescription for an ADHD treatment; 93 (11.1%) for an anticonvulsant; 315 (37.6%) for an antidepressant; two (0.2%) for an antiparkinsonian; 24 (2.9%) for an antipsychotic; and 446 (53.3%) for a sedative-anxiolytic. A total of 100 (11.9%) had prescriptions for two or more classes of drug.

There were significant differences between the study cohort and those with diagnoses by gender (χ^2 ^= 38.9, P < 0.001) and military specialty (χ^2 ^= 3.44, P = 0.025), and between the study cohort and those with prescriptions by gender (χ^2 ^= 115, P < 0.001), military specialty (χ^2 ^= 16.3, P ≤ 0.001), and age category (χ^2 ^> 1000, P < 0.001). Between the study cohort and those with either diagnosis or prescription, there were significant differences by gender (χ^2 ^= 124, P < 0.001), military specialty (χ^2 ^= 17.4, P < 0.001), and age category (χ^2 ^= 14.3, P < 0.001). Women had over twice the odds of having either diagnosis or prescription (OR = 2.48, 95% CI 2.11–2.93). Among those with any deployment history, the odds of either diagnosis or prescription was higher, but this difference was not statistically significant (OR = 1.10, 95% CI 0.975–1.25).

## Discussion

This is the first study to assess the prevalence of contraindications to mefloquine use among U.S. service members deployed to a malarious area and for whom long-term chemoprophylaxis against *Plasmodium vivax *is indicated. This study found a high prevalence of contraindications to the safe use of this drug in this population overall, and a statistically significant elevation in the prevalence of contraindications among females.

This study was performed to determine the prevalence of contraindications to mefloquine use in a deployed U.S. military cohort, and as such has a number of limitations that preclude its generalizability to the non-military U.S. population. This study examined a highly biased sample of young adults (93.8% who were under the age of 40), self-selected for military service from the U.S. general population and subject to considerable selection pressures from commissioning, enlistment, or periodic health screenings. These pressures would ordinarily result in this population being generally more healthy than members of the U.S. civilian population, and would ordinarily be expected to reduce the prevalence of health conditions incompatible with continued military service and deployment.

Interestingly, this bias, which is commonly referred to as the "healthy-warrior effect" [11] may not be applicable in this study population for those health conditions consistent with a contraindication to mefloquine use. The prevalences of both acute and chronic mental health diagnoses are high among cohorts of previously deployed service members [12,13], although in this study population a statistically significant difference was not found in the prevalence of contraindications among those with and without such deployment history.

Despite the substantial differences in health histories and demographics between studies, this study finds a similar overall rate of contraindication to mefloquine use as a prior prospective study in the general population, in which 9% of 2,389 civilian travelers with available medical histories presenting to a travel medicine clinic were evaluated [14]. Similar to the present study, this prior study found a history of psychiatric contraindication in 3.5% of the group. Unlike the present study, in which neurological contraindications were rare, these were present in 5.7% of the prior study group. This difference likely reflects the incompatibility of most neurological conditions with military service.

This study employed medical surveillance databases to identify an arbitrary set of ICD-9CM diagnosis codes defining a medical contraindication to mefloquine use. The diagnosis codes were selected based on the clinical judgment of the study authors. This methodology reflects the clinical uncertainty faced by individual healthcare providers in interpreting the mefloquine package insert. Of the listed contraindications to mefloquine use, the terms "major psychiatric disorders" and "history of convulsions" leave significant room for interpretation. In this study, a liberal interpretation of neurological disorder, and a relatively conservative interpretation of psychiatric disorder were employed. This had the effect of including and excluding, respectively, many diagnoses which individual providers might or might not consider a contraindication.

The use of pharmacosurveillance databases to infer the presence of a contraindicating medical condition is reasonable. Previous studies [8] have shown significant correlation between psychiatric diagnosis and psychoactive drug prescription, and the dispensing of a drug in this class in the absence of a diagnostic encounter likely represents continued therapy without documentation of follow-up treatment.

These findings underscore that caution must be exercized by healthcare providers in prescribing mefloquine to deploying U.S. military personnel, and lend strong support to policies prohibiting the practice of mass-prescribing mefloquine without an individualized review of medical records. Given the significant prevalence of its contraindications, a careful review and screening of available medical records (including available pharmacy records) and thorough counseling prior to prescribing and dispensing mefloquine to U.S. military personnel is strongly indicated. The use of existing medical and pharmacosurveillance databases to populate automated decision-support systems to assist clinicians in identifying personnel with possible contraindications may improve the sensitivity of such review and screening. The development of such systems would require that the ICD-9CM codes and medications deemed consistent with a contraindication to mefloquine use be formally defined.

This study also highlights the importance of investing in the further development and testing of alternative anti-malaria drugs which retain the advantages in compliance and convenience of the weekly dosing schedule of mefloquine. For U.S. service members in whom mefloquine in contraindicated, tafenoquine may be a promising alternative [15], both for its schizonticidal activity and its potential use as terminal prophylaxis against *Plasmodium vivax *infection [16].

Although not sharing the particular contraindications of mefloquine, tafenoquine carries a significant risk of haemolysis among those with glucose-6-phosphate dehydrogenase (G6PD) deficiency syndrome. A recent study suggests that the prevalence of this contraindication among deploying U.S. military populations is lower than that of contraindications to mefloquine found in the present study, with 2.5% of males and 1.6% of females deficient for G6PD [17].

In light of this study's findings, upon U.S. Food and Drug Administration licensure and upon the completion of studies confirming its safety and suitability for long-term administration, tafenoquine may prove to be a viable option to replace mefloquine for routine use in the U.S. military. Universal screening for G6PD deficiency upon entry to U.S. military service may be a prudent means of ensuring the safe administration of this drug and aiding in the pre-selection of individuals most suited for long-term deployment to malarious areas.

## Abbreviations

ADHD : Attention-Deficit Hyperactivity Disorder; CI: Confidence Interval; DMSS: Defense Medical Surveillance System; G6PD: Glucose-6-Phosphate Dehydrogenase; ICD9-CM: International Classification of Diseases, Ninth Revision, Clinical Modification; OR: Odds Ratio; PDTS: Pharmacy Data Transaction System

## Competing interests

The author(s) declare that they have no competing interests.

## Authors' contributions

JBC conceived of the study. PPP coordinated acquisition of DMSS data and assisted in analysis. RLN integrated PDTS, and DMSS data, performed the statistical analyses, and drafted the manuscript. All authors assisted in interpreting the findings and reviewing and editing the manuscript.

## Disclaimer

The opinions expressed in this paper are those of the authors and do not reflect the official policies or positions of the Department of the Army or the Department of Defense.
